# Cost-effectiveness of new antiviral treatments for non-genotype 1 hepatitis C virus infection in China: a societal perspective

**DOI:** 10.1136/bmjgh-2020-003194

**Published:** 2020-11-27

**Authors:** Xia Wei, Jingyu Zhao, Li Yang

**Affiliations:** Department of Health policy and management, School of Public Health, Peking University, Beijing, China

**Keywords:** health economics, health insurance, viral hepatitis, other study design

## Abstract

**Objective:**

This study aimed to estimate the cost-effectiveness of direct-acting antivirals (DAAs) among patients with non-genotype 1 for the eradication of hepatitis C virus (HCV) infection in China.

**Methods:**

A decision-analytic Markov model was developed to estimate the lifetime costs, quality-adjusted life years (QALYs) and incremental cost-effectiveness ratios (ICERs) for DAAs and pegylated interferon plus ribavirin (PEG-RBV) from a societal perspective. The model inputs were derived from the literature, a patient survey, HCV expert opinions and a specialised drug price database available in China. Sensitivity analysis was conducted to evaluate the model robustness and calculate reasonable prices of DAAs.

**Results:**

For patients infected with HCV genotype 2, the pan-genotypic regimen sofosbuvir/velpatasvir (SOF/VEL) was the most cost-effective strategy compared with PEG-RBV, with an ICER of US$5653/QALY. For genotype 3, the combination of sofosbuvir plus daclatasvir (SOF-DCV) was the most cost-effective approach, with an ICER of US$3314/QALY. All DAA regimens for genotype 6 were cost-saving, and sofosbuvir plus ribavirin (SOF-RBV) was the optimal regimen. One-way sensitivity analysis demonstrated that the ICERs were most sensitive to the utility values, discount rate and drug costs. Probabilistic sensitivity analysis indicated that using a threshold equal to one time the gross domestic product (GDP) per capita in China (US$9769/QALY, 2018), the probability of SOF/VEL, SOF-DCV and SOF-RBV being cost-effective was 58%, 83% and 71% for genotype 2, 3 and 6, respectively. Threshold analysis showed that the price of DAAs should be reduced by some degree to achieve better affordability.

**Conclusions:**

DAAs were cost-effective compared with traditional treatments. A reasonable reduction in the price of DAAs will increase drug affordability and is of great significance as a global strategy to eradicate viral hepatitis.

**Key questions**

**What is already known?**

- The direct-acting antivirals (DAAs) have dramatically improved the efficacy and reduced the burden of adverse events, making it possible to eradicate hepatitis C virus (HCV) globally.
- The international and national studies have proved the cost-effectiveness of some DAAs compared with pegylated interferon plus ribavirin (PEG-RBV) for patients with genotype 1 from a payer perspective.

**What are the new findings?**

- This study aimed to estimate the cost-effectiveness of all DAAs compared with PEG-RBV for patients with non-genotype 1 from a societal perspective in the Chinese setting.
- This was the first evaluation to quantitatively compare pan-genotypic regimen sofosbuvir/velpatasvir (SOF/VEL) with other DAAs or PEG-RBV especially for patients with non-genotype 1 in China considering limited access of gene testing in underdeveloped areas.
- Threshold analysis was used to calculate reasonable prices of different DAAs to achieve better economic efficiency when comparing with optimal DAA regimen in each genotype.

**What do the new findings imply?**

- For patients with non-genotype 1 in China, DAAs were more cost-effective than PEG-RBV, and produced better health outcomes and higher quality of life.
- More importantly, reasonable reduction in the price of DAAs will increase drug affordability and is of great significance for the global strategy to eradicate viral hepatitis, especially for the pan-genotypic regimen SOF/VEL that can simplify the clinical pathway of HCV infection.

## Introduction

Hepatitis C virus (HCV) infection has become a global public health issue, and the relevant long-term complications, such as liver cirrhosis or hepatocellular carcinoma, have imposed large health and economic burdens on patients. There are around 71 million patients infected with HCV worldwide, leading to approximately 399 000 deaths each year.^1^ In China, the incidence of HCV infection increased sharply in the last decade, and the estimated number of patients reached 9.8 million in 2015.^1^ However, in the same period, the diagnosis rate was only 2%, and the treatment rate was less than 1% in China, far below the global average.^2 3^ Liver cirrhosis and hepatocellular carcinoma are the leading causes of death from HCV. The mortality rate of liver cirrhosis caused by HCV in China was 0.2 per 100 000 people, and that of hepatocellular carcinoma was 0.4 per 100 000 people in 2016.^4^

The primary aim of HCV infection treatment is to achieve sustained virologic response (SVR), which can significantly reduce the risk of progression to liver cirrhosis or hepatocellular carcinoma.^5^ Pegylated interferon plus ribavirin (PEG-RBV) was the standard of care in China before the introduction of direct-acting antivirals (DAAs) in 2017.^6^ Compared with the suboptimal efficacy and severe adverse events of PEG-RBV, DAAs have drastically decreased morbidity and mortality attributable to HCV infection. However, patients still have limited access to such treatments in China since several DAAs, such as asunaprevir, daclatasvir, sofosbuvir, sofosbuvir/ledipasvir, elbasvir/grazoprevir and sofosbuvir/velpatasvir (SOF/VEL), were not approved through priority review procedure until 2017. In addition, DAAs were more costly than traditional PEG-RBV therapy, and PEG-RBV was the only treatment on the national drug reimbursement list until the end of 2019.

Although the most prevalent HCV genotype in China is genotype 1, patients with non-genotype 1 still account for 43.2%.^6^ Unlike the diverse options for patients with genotype 1, they can only be treated with limited types of DAAs. Among those patients, genotypes 2 (15.8%), 3 (8.7%) and 6 (5.7%) are the majority, as well as genotypes 4 and 5 with nearly no report.^6 7^ Significant regional differences exist in the genotype distribution. Genotypes 2 and 3 are more prevalent in western China, and genotype 6 is more prevalent in southern China.^6^

At present, scant evidence is available on the cost-effectiveness of DAAs for patients with non-genotype 1. Hence, this study aimed to estimate the cost-effectiveness of all treatment regimens for patients with non-genotype 1 and provide insights for the eradication of viral hepatitis in China and globally.

## Methods

### Model structure and assumptions

**Figure 1:**
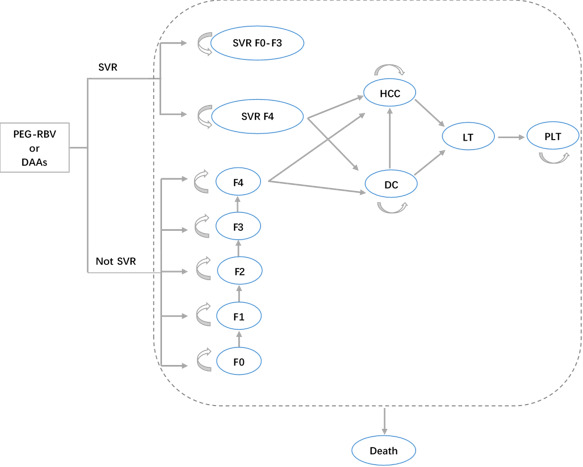
The structure of the decision-analytic Markov model. DAAs, direct-acting antivirals; DC, decompensated cirrhosis; F0–F4, METAVIR fibrosis states; HCC, hepatocellular carcinoma; LT, liver transplantation; Not SVR, not achieving sustained virologic response; PEG-RBV, pegylated interferon plus ribavirin; PLT, post liver transplantation; SVR, sustained virologic response; SVR F0–F3, patient in F0–F3 states achieving SVR; SVR F4, patient in F4 state achieving SVR.

A decision-analytic Markov model was developed based on the results of previous studies.^8–10^ Fifteen exclusive health states were addressed, as shown in figure 1: METAVIR liver fibrosis states (F0—no fibrosis, F1—portal fibrosis without septa, F2—portal fibrosis with few septa, F3—numerous septa without cirrhosis and F4—compensated cirrhosis), liver fibrosis states achieving SVR (SVR F0–F4), decompensated cirrhosis (DC), hepatocellular carcinoma (HCC), liver transplantation (LT), post liver transplantation (PLT) and death. The model comprised the stages of treatment and disease progression. Patients received different treatment regimens when entering the model. Subsequent stages were determined according to their initial health state and treatment outcome. Annual cycles and lifetime horizon were adopted. The key assumptions of the study were as follows^11–14^: (1) spontaneous elimination of the virus was not considered; (2) patients received treatment only once, without considering further treatment after treatment failure or recurrence; (3) treatment compliance was 100%; (4) patients in F0–F3 states would no longer experience disease progression after achieving SVR, while patients in F4 state with SVR and all patients without SVR would enter a stage of disease progression.

### Characteristics of patients

The target population was treatment-naive patients infected with HCV genotypes 2, 3 and 6. The model followed up a hypothetical cohort of 10 000 patients for each of the three genotypes. Baseline characteristics were obtained from a cross-sectional observational study across China,^7^ where the median ages of patients infected with HCV genotypes 2, 3 and 6 were 48, 38 and 35, respectively. Therefore, the study assumed that patients entered the model at the age of 50, 40 and 35 for genotypes 2, 3 and 6, respectively. Based on the same study, the proportion of males was 50.80%, 75.80% and 66.70% for patients infected with HCV genotypes 2, 3 and 6, respectively.^7^ According to another Chinese study, which observed patients with HCV for 20 years, the baseline distributions of METAVIR fibrosis states were 0.80% (F0), 45.50% (F1), 41.30% (F2), 9.90% (F3) and 2.50% (F4), respectively.^15^

### Treatment regimens and clinical inputs

Treatment regimens for each genotype were defined following current clinical guidelines and proposed indications for DAAs in China.^6^ For patients infected with HCV genotype 2, four treatment regimens were estimated: PEG-RBV for 24 weeks, and sofosbuvir plus ribavirin (SOF-RBV), sofosbuvir plusdaclatasvir (SOF-DCV) and SOF/VEL for 12 weeks. For genotype 3, PEG-RBV and SOF-RBV for 24 weeks, and SOF-DCV and SOF/VEL for 12 weeks were considered. For genotype 6, PEG-RBV for 48 weeks, and SOF-RBV, SOF-DCV and SOF/VEL for 12 weeks were considered. The primary efficacy measurement in the model was SVR, defined as an undetectable HCV RNA level 12 weeks after the end of the treatment.^6^

Available treatment regimens and the corresponding SVR for patients with non-cirrhosis (F0–F3) and patients with cirrhosis (F4) were reported in online supplemental table 1. The SVRs of PEG-RBV and SOF-DCV were derived from systematic reviews or meta-analysis.^16–22^ The SVRs of SOF-RBV and SOF/VEL were derived from three published clinical trials in Asian or Chinese settings.^21–23^ Due to limited data availability, SVR for patients with cirrhosis infected with genotype 6 was assigned the same setting as that of patients with non-cirrhosis.

10.1136/bmjgh-2020-003194.supp1 Supplementary data

### Transition probabilities

Since large-sample epidemiological research is rare in China, this study used transition probabilities from other countries. Transition probabilities were assumed comparable among the three genotypes.^14 24–26^ The probabilities of liver fibrosis progression between F0 and F4 states were derived from a meta-analysis using Markov maximum likelihood estimation.^27^ Patients in F4 state could progress to DC and HCC, and the respective probabilities were obtained from Dienstag *et al*.^28^ Patients with DC also experienced the transition to HCC.^28^ Only patients with DC and HCC would receive a liver transplant, and the corresponding probabilities were derived from Townsend *et al*^29^ and Planas *et al.*^30^ After achieving SVR, patients in F4 state could progress to advanced liver disease but typically experience a lower risk than those who did not achieve SVR. The mortality rates associated with DC, HCC, LT and PLT, higher than the general mortality, were derived from the literature.^31–34^ Age-specific all-cause mortality rates were extracted from the life tables of the WHO member states.^35^ It was assumed that F0–F3 patients had the same mortality rates as the general population after achieving SVR. F4 patients achieving SVR and F0–F4 patients who did not achieve SVR had 1.4^36^ and 2.37^37^ times the background mortality, respectively. The mortality rates were not adjusted for patients older than 65 due to the significantly higher general mortality.

### Cost inputs

The societal perspective was adopted in this study. All costs were converted to US$ using official exchange rates of 2018 (US$1=￥6.62) and inflated to 2018 prices using the China Consumer Price Index. All costs were listed in online supplemental table 1.

The direct medical costs consisted of the drug, monitoring and annual liver-related health state costs. Drug costs were regimen-specific, with different drug prices and treatment duration. Additionally, drug prices were obtained from a local database^38^ reporting the price of drugs in each province of China. Due to the societal perspective adopted, this study used the median prices of different provinces paid by patients only or by patients and payers together with drug reimbursement. According to the latest guidelines for HCV infection in China,^6^ patients should undergo genotyping and liver fibrosis tests to assess the severity of liver disease before treatment, as well as routine surveillance to monitor the efficacy and safety of the cure during treatment. Routine surveillance was carried out every 3 months during treatment, including plasma HCV RNA, complete blood count and liver ultrasound, among others; the number of times patients were tested varied depending on the treatment duration. Based on local charges of tests and consultation with HCV experts, the costs of genotyping, liver fibrosis test and routine surveillance were set at US$63, US$21 and US$302, respectively. Annual liver-related health state costs associated with F0–F4, DC and HCC were obtained from a study addressing the main hospitals in eight cities in Mainland China.^39^ For LT and PLT, costs were derived from a disease burden study for Chinese liver transplant patients.^40^ Patients in F0–F3 states with SVR would not incur direct medical costs any more, and patients in F4 state with SVR need less health resource utilisation, 0.709 times of those in F4 state without SVR.^41^

Due to limited research on direct non-medical and indirect costs of patients with HCV in China, data on these aspects were obtained from a patient survey conducted in Tianjin for centralised and standardised patient management. Adult patients with HCV receiving PEG-RBV or DAAs in the past few years were invited by telephone to participate in the survey. A total of 155 patients receiving PEG-RBV and 145 patients receiving DAAs were investigated in 2018–2019. Based on the characteristics of the HCV treatment, the direct non-medical costs were the transportation and nutrition costs of patients and their family members, and the indirect costs were the productivity loss of patients and their family members. The human capital approach was used to calculate the productivity loss based on working time lost and the per capita disposable income of China in 2018. Combined with treatment duration, the direct non-medical costs and indirect costs were calculated for each regimen.

### Utility inputs

Utility inputs consisted of three parts, as shown in online supplemental table 1. Since Chinese utility values were not available, this study used data from the international literature. Utility values of chronic HCV states were derived from systematic reviews of the quality of life of patients with HCV.^42 43^ Patients would experience a utility decrease during treatment due to treatment-related adverse events, with utility decreasing by 0.11 under PEG-RBV and by 0.03 under DAAs treatment.^44^ Utility values after achieving SVR for F0–F4 states were obtained from Wright *et al*^45^

### Model analysis

The proposed model was developed in Microsoft Excel 2016 software (Microsoft). The future costs and QALYs were discounted at an annual rate of 5%.^46^ The willingness-to-pay was set at US$9769/QALY adopting the threshold of one time the GDP per capita of 2018 in China. Lifetime costs, quality-adjusted life years (QALYs) and incremental cost-effectiveness ratios (ICERs) were illustrated as efficiency frontiers.

One-way sensitivity analysis was conducted to assess the influence of changes in individual parameters on the model results. The costs, SVR, transition probabilities, utilities and discount rate were tested under the range defined in the inputs table. The 95% CI of each parameter was used as the lower and upper values for the one-way sensitivity analysis. In case, the 95% CI was not available, the parameter varied by ±25%. In addition, the discount rate ranged between 0% and 8%. The results were shown as tornado diagrams.

Monte Carlo simulation was performed in probabilistic sensitivity analysis to test the robustness of the model results when all parameters varied simultaneously. A total of 1000 Monte Carlo iterations were run by repeatedly sampling from the distributions assigned to all the uncertain parameters (gamma distribution for costs, and beta or uniform distribution for SVR, utilities and transition probabilities). The results were shown as cost-effectiveness acceptability curves, which reflected the probabilities of the treatment regimens being cost-effective at different willingness-to-pay thresholds.

Additionally, threshold analysis was performed to improve affordability for patients. Keeping all the other parameters constant, the study estimated the threshold price, where the treatment regimen showed equal economic efficiency compared with the optimal DAA regimen for each genotype.

### Patient and public involvement

This study did not involve any direct patient and public involvement.

## Results

### Base-case analysis

**Figure 2:**
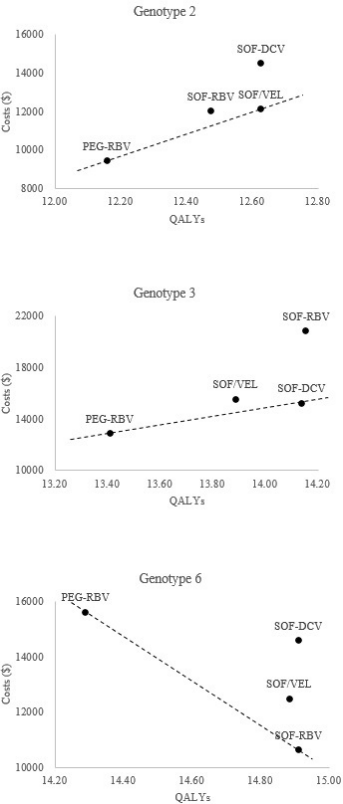
Efficiency frontiers of different regimens in three genotypes. PEG-RBV, pegylated interferon plus ribavirin; SOF-DCV, sofosbuvir plus daclatasvir; SOF-RBV, sofosbuvir plus ribavirin; SOF/VEL, sofosbuvir/velpatasvir; QALYs, quality-adjusted life years.

The base-case results were presented in table 1 and figure 2. For patients infected with HCV genotype 2, DAAs gained more QALYs compared with PEG-RBV but with higher costs. The efficiency frontier consisted of PEG-RBV and SOF/VEL. SOF-RBV and SOF-DCV were above the efficiency frontier due to lower effectiveness or higher costs. The ICERs of SOF/VEL, SOF-RBV and SOF-DCV versus PEG-RBV were US$5653, US$7999 and US$10 680/QALY, respectively. SOF/VEL was the most cost-effective regimen and reduced the greatest cumulative probabilities of DC and HCC by 4.29% and 3.22%, respectively. The results were similar for genotype 3. SOF-DCV was the most cost-effective regimen, with the ICER of US$3314/QALY, which reduced the cumulative probabilities of DC and HCC by 8.09% and 6.26%, respectively. Three DAA regimens for genotype 6 were cost-saving; SOF-RBV was the most cost-saving approach due to its outstanding health benefits.

**Table 1:** Cost-effectiveness of different regimens in the Chinese setting

Treatment regimen	DC (%)	HCC (%)	QALYs	Costs (US$)	Incremental QALYs	Incremental costs (US$)	ICER (US$/QALY)
Genotype 2							
PEG-RBV (reference)	4.47	3.62	12.1557	9470	–	–	–
SOF/VEL	0.18	0.39	12.6250	12 123	0.4693	2653	5653
SOF-RBV	1.86	1.66	12.4721	12 001	0.3164	2531	7999
SOF-DCV	0.18	0.39	12.6250	14 482	0.4693	5012	10 680
Genotype 3							
PEG-RBV (reference)	9.50	7.55	13.4059	12 861	–	–	–
SOF-DCV	1.41	1.29	14.1355	15 279	0.7296	2418	3314
SOF/VEL	4.58	3.82	13.8841	15 541	0.4782	2680	5604
SOF-RBV	1.23	1.23	14.1528	20 834	0.7469	7973	10 675
Genotype 6							
PEG-RBV (reference)	7.41	6.02	14.2874	15 630	–	–	–
SOF-RBV	0.26	0.55	14.9081	10 635	0.6207	−4995	−8047
SOF/VEL	0.61	0.83	14.8810	12 493	0.5936	−3137	−5285
SOF-DCV	0.26	0.55	14.9081	14 598	0.6207	−1032	−1663

DC, decompensated cirrhosis; HCC, hepatocellular carcinoma; ICER, incremental cost-effectiveness ratio; PEG-RBV, pegylated interferon plus ribavirin; QALYs, quality-adjusted life years; SOF-DCV, sofosbuvir and daclatasvir; SOF-RBV, sofosbuvir plus ribavirin; SOF/VEL, sofosbuvir/velpatasvir.

### Sensitivity analysis

**Figure 3:**
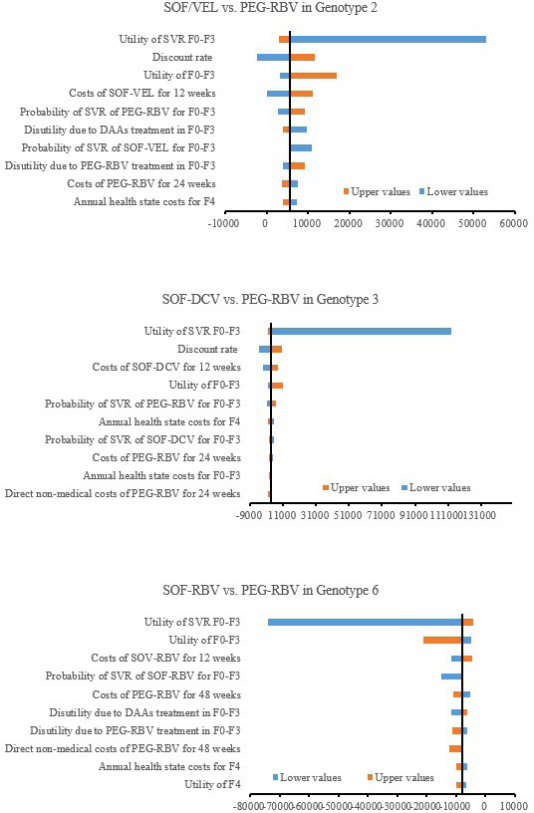
Tornado diagrams of the optimal regimen compared with PEG-RBV in three genotypes. DAAs, direct-acting antivirals; F0–F4, METAVIR fibrosis states; PEG-RBV, pegylated interferon plus ribavirin; SVR, sustained virologic response; SOF-DCV, sofosbuvir plus daclatasvir; SOF-RBV, sofosbuvir plus ribavirin; SOF/VEL, sofosbuvir/velpatasvir.

In one-way sensitivity analysis, the most cost-effective regimen compared with PEG-RBV was analysed for each genotype. The tornado diagrams of the 10 most sensitive parameters were shown in figure 3. The ICERs were most sensitive to the utility of patients in F0–F3 states after achieving SVR, while the other factors, including the discount rate, drug costs and SVR, were slightly different. For genotypes 2 and 3, the adjustment of some parameters, such as the discount rate, could make the optimal regimen no longer cost-effective. For genotype 6, changes in the parameters had no fundamental influence on base-case results.

**Figure 4:**
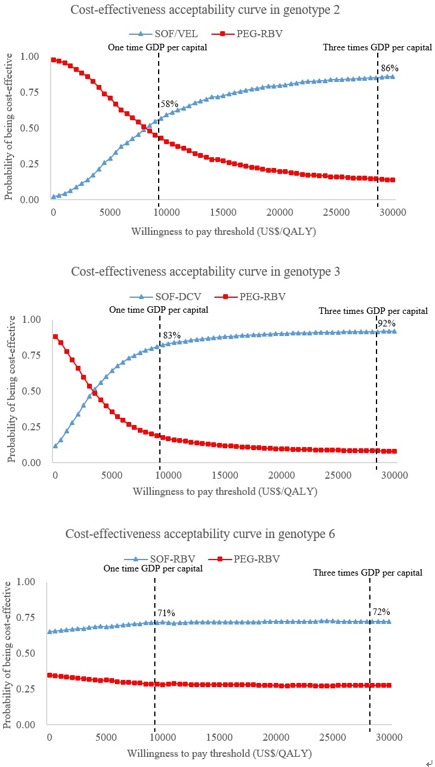
Cost-effectiveness acceptability curve in three genotypes. GDP, gross domestic product; PEG-RBV, pegylated interferon plus ribavirin; QALY, quality-adjusted life year; SOF-DCV, sofosbuvir plus daclatasvir; SOF-RBV, sofosbuvir plus ribavirin; SOF/VEL, sofosbuvir/velpatasvir.

The probability sensitivity analysis also used the most cost-effective regimen for comparison. The cost-effectiveness acceptability curves showed that under a threshold of US$9769/QALY, the probability of SOF/VEL, SOF-DCV and SOF-RBV being cost-effective was 58%, 83% and 71% for genotypes 2, 3 and 6, respectively, as shown in figure 4. If the threshold was increased to three times the Chinese GDP per capita (US$29 307), the corresponding probability would increase to 86%, 93% and 72%, respectively.

### Threshold analysis

We chose the optimal DAA regimen for comparison in threshold analysis for each genotype. For genotype 2, the price reduction needed for sofosbuvir and daclatasvir to achieve the same economic efficiency as the optimal regimen SOF/VEL was 8% and 41%, respectively. To achieve cost-saving for all regimens for genotype 2, sofosbuvir, daclatasvir and SOF/VEL need a price reduction of 28%, 63% and 25%, respectively. For genotype 3, the price reduction of sofosbuvir and SOF/VEL should be 31% and 10%, respectively, compared with the optimal regimen SOF-DCV. To achieve further cost-saving, sofosbuvir and SOF/VEL need to cut price by 45% and 25%, respectively. Since all regimens for genotype 6 were cost-saving, sofosbuvir, daclatasvir and SOF/VEL need to reduce their price by 31%, 31% and 20% to achieve the same economic efficiency as SOF-RBV. The threshold analysis results were reported in table 2.

**Table 2:** Threshold analysis results in three genotypes

Genotype	Comparator	DAA drugs	Threshold price (US$)	Price reduction (%)
Genotype 2	SOF/VEL	Sofosbuvir	2723	8
		Daclatasvir	391	41
	To be cost-saving	Sofosbuvir	2127	28
		Daclatasvir	247	63
		Sofosbuvir/velpatasvir	2622	25
Genotype 3	SOF-DCV	Sofosbuvir	2055	31
		Sofosbuvir/velpatasvir	3141	10
	To be cost-saving	Sofosbuvir	1642	45
		Sofosbuvir/velpatasvir	2613	25
Genotype 6	SOF-RBV	Sofosbuvir	2056	31
		Daclatasvir	457	31
		Sofosbuvir/velpatasvir	2798	20

DAA, direct-acting antiviral; SOF-DCV, sofosbuvir plus daclatasvir; SOF-RBV, sofosbuvir plus ribavirin; SOF/VEL, sofosbuvir/velpatasvir.

## Discussion

Few studies investigated the cost-effectiveness of treatment regimens for patients infected with non-genotype 1 HCV, especially genotype 6, in China.^47 48^ This study assessed the cost-effectiveness of all available DAAs and PEG-RBV for patients infected with genotypes 2, 3 and 6 in China. Base-case results demonstrated that SOF/VEL and SOF-RBV were more cost-effective than PEG-RBV, while the ICER of SOF-DCV exceeded the willingness-to-pay threshold for genotype 2. For genotype 3, SOF-DCV and SOF/VEL were more cost-effective than PEG-RBV, while SOF-RBV was not cost-effective. SOF-RBV, SOF/VEL and SOF-DCV were all economically dominant relative to PEG-RBV for genotype 6, as the treatment duration of genotypes 2 and 3 with PEG-RBV was only half than that of genotype 6. Sensitivity analysis demonstrated that the base-case results were robust. More importantly, the prices of DAA drugs need to be reduced by a range of 8%–63% compared with the most cost-effective DAA regimen for each genotype.

Several cost-effectiveness analyses have been conducted in China. However, most studies focused on patients infected with genotype 1 HCV.^11 39 49^ Among these studies, Chen H *et al*^11^ and Chen GF *et al*^39^ compared DAAs with PEG-RBV for genotype 1; however, their results might have potential limitations in the Chinese setting because information on drug costs was obtained from other countries (DAAs were not available in China at that time). Another study evaluated the pan-genotypic SOF/VEL with other DAAs, indicating that a lower price of SOF/VEL would make it more cost-effective; however, the generality of this study’s findings was limited because only genotype 1 was considered.^49^ Wu *et al* are the only researchers who evaluated the cost-effectiveness of SOF-RBV and SOF-DCV for Chinese patients with non-genotype 1, showing that SOF-RBV was a cost-effective alternative for genotypes 2 and 3 and a cost-saving alternative for genotype 6 relative to PEG-RBV.^14^ However, the pan-genotypic SOF/VEL, which simplified the treatment, was not included in their study. To the authors’ knowledge, no cost-effectiveness study included all available DAAs for the treatment of patients with non-genotype 1 in China. Hence, this study is likely to represent the first economic evaluation in this field.

The societal perspective was adopted in several studies mainly conducted in the USA. Two American studies compared the cost-effectiveness of DAAs, DAAs plus PEG-RBV and PEG-RBV using a similar Markov model.^50 51^ The results showed that SOF-based treatment was cost-effective, while affordability was to be considered. However, only drug-related and liver-disease-related costs were included, and only patients with genotype 1 were addressed. Another study estimating DAA regimens with PEG-RBV indicated that the novel treatments were cost-effective compared with standard care for genotype 1 and probably genotype 3 but not for patients with genotype 2 in the American setting.^25^ Similarly, only direct medical costs were included, while indirect costs due to productivity loss or non-medical costs were not calculated. When the costs of absenteeism, presenteeism and patient/caregiver time were included, the DAAs were found to be cost-saving relative to no treatment in another American study.^52^ SOF-RBV was cost-effective relative to PEG-RBV in patients with genotype 2, considering costs of the productivity loss in the Japanese scenario,^53^ in line with our results.

DAAs offer more effective, shorter and better-tolerated treatment options for patients; hence, a comparison among DAAs is needed. When compared with the optimal DAA regimen for each genotype, other DAAs need a price reduction to achieve equal economic efficiency. Since the use of SOF/VEL creates an opportunity to simplify the care pathway by removing the need for genotyping, extra direct non-medical costs such as transportation costs can be saved.^49^ This aspect is of particular significance for primary healthcare institutions, where genotyping tests cannot be accessed to manage patients with HCV. In this study, SOF/VEL was found to be cost-effective for all genotypes, while it needed a price reduction of 10% and 20% compared with SOF-DCV in genotype 2 and SOF-RBV in genotype 6, respectively.

The present analysis has several limitations. First, some SVR rates were derived from separate clinical trials due to the scarcity of head-to-head trials comparing those treatment regimens, which may result in biased results. Second, transition probabilities were derived from international research in the absence of domestic information sources. Future studies should update the analysis when these data are available in China. Third, utility values from other countries were assigned to health states due to the unavailability of Chinese-specific utility data, which may affect the accuracy of our results. In addition, the sample size of our patient survey needs to be increased to improve the generality of direct non-medical costs and indirect costs. Finally, our results may differ from the final discounted prices after price negotiation in 2019 as the drug costs were obtained from market prices, and some newly approved regimens, such as glecaprevir/pibrentasvir, should be considered in the future.

## Conclusion

DAAs were cost-effective compared with traditional treatments for Chinese patients with non-genotype 1 HCV, but the ICERs of DAAs were different. To globally eradicate viral hepatitis by 2030, DAAs’ prices need to be reduced by some degree to improve economic efficiency and increase the affordability of these drugs, especially in developing countries.
