# Supplementary material for: Cost-effectiveness of new antiviral treatments for non-genotype 1 hepatitis C virus infection in China: a societal perspective
Source: BMJ Glob Health. 2020 Nov 27;5(11):e003194. doi: 10.1136/bmjgh-2020-003194 (PMC7703443; doi:10.1136/bmjgh-2020-003194)
Supplement: Supplementary data [file bmjgh-2020-003194supp001.pdf]

Supplemental table 1. Model inputs

Parameters	Base-case value (range)	Distribution (a, b)	Reference
**Probability of SVR**			
Genotype 2-without cirrhosis			
PEG-RBV for 24 weeks	0.819（0.769-0.862）	Beta (213.9, 47.2)	[18]
SOF-RBV for 12 weeks	0.928（0.830-0.970）	Beta (47.7, 3.7)	[22]
SOF-DCV for 12 weeks	1.000（0.930-1.000）	Uniform (0.9, 1.0)	[20]
SOF/VEL for 12 weeks	1.000（0.940-1.000）	Uniform (0.9, 1.0)	[23]
Genotype 2-with cirrhosis			
PEG-RBV for 24 weeks	0.621（0.497-0.734）	Beta (39.1, 23.9)	[18]
SOF-RBV for 12 weeks	0.875（0.830-0.970）	Beta (74.2, 10.6)	[22]
SOF-DCV for 12 weeks	1.000（0.930-1.000）	Uniform (0.9, 1.0)	[20]
SOF/VEL for 12 weeks	1.000（0.940-1.000）	Uniform (0.9, 1.0)	[23]
Genotype 3-without cirrhosis			
PEG-RBV for 24 weeks	0.705（0.639-0.765）	Beta (141.9, 59.3)	[18]
SOF-RBV for 24 weeks	0.971（0.900-0.980）	Beta (64.7, 1.9)	[22]
SOF-DCV for 12 weeks	0.974（0.932-0.998）	Beta (86.0, 2.3)	[16]
SOF/VEL for 12 weeks	0.860（0.760-0.920）	Beta (61.3, 10.0)	[23]
Genotype 3-with cirrhosis			
PEG-RBV for 24 weeks	0.602（0.558-0.644）	Beta (301.2, 199.0)	[18]
SOF-RBV for 24 weeks	0.875（0.656-1.000）	Beta (11.6, 1.7)	[22]
SOF-DCV for 12 weeks	0.625（0.449-0.786）	Beta (19.2, 11.5)	[16]
SOF/VEL for 12 weeks	0.860（0.760-0.920）	Beta (61.3, 10.0)	[23]
Genotype 6-without cirrhosis			
PEG-RBV for 48 weeks	0.800（0.730-0.850）	Beta (135.8, 33.9)	[17]
SOF-RBV for 12 weeks	1.000（0.690-1.000）	Uniform (0.7, 1.0)	[21]
SOF-DCV for 12 weeks	1.000（0.750-1.000）	Uniform (0.8, 1.0)	[19]
SOF/VEL for 12 weeks	0.990（0.940-1.000）	Beta (40.8, 0.4)	[23]
Genotype 6-with cirrhosis			
PEG-RBV for 48 weeks	0.800（0.730-0.850）	Beta (135.8, 33.9)	[17]
SOF-RBV for 12 weeks	1.000（0.690-1.000）	Uniform (0.7, 1.0)	[21]
SOF-DCV for 12 weeks	1.000（0.750-1.000）	Uniform (0.8, 1.0)	[19]
SOF/VEL for 12 weeks	0.990（0.940-1.000）	Beta (40.8, 0.4)	[23]
**Transition probabilities**			

**Natural history of HCV**			
F0→F1	0.117 (0.104-0.130)	Beta (274.6, 2072.8)	[27]
F1→F2	0.085 (0.075-0.096)	Beta (230.3, 2478.8)	[27]
F2→F3	0.120 (0.109-0.133)	Beta (337.9, 2478.2)	[27]
F3→F4	0.116 (0.104-0.129)	Beta (292.3, 2227.8)	[27]
F4→DC	0.039 (0.029-0.049)	Beta (59.0, 1454.5)	[28]
F4→HCC	0.024 (0.018-0.030)	Beta (60.0, 2438.6)	[28]
DC→HCC	0.024 (0.018-0.030)	Beta (60.0, 2438.6)	[28]
DC→LT	0.023 (0.017-0.029)	Beta (60.0, 2549.9)	[29]
DC→Death	0.104 (0.078-0.130)	Beta (55.0, 473.6)	[31-33]
HCC→LT	0.040 (0.030-0.050)	Beta (59.0, 1415.2)	[31-33]
HCC→Death	0.520 (0.390-0.650)	Beta (29.0, 26.8)	[31-33]
LT→Death	0.194 (0.146-0.243)	Beta (49.3, 205.0)	[34]
PLT→Death	0.049 (0.037-0.061)	Beta (58.4, 1133.5)	[34]
Achieving SVR			
SVR F4→DC	0.003 (0.002-0.004)	Beta (61.3, 20364.8)	[28]
SVR F4→HCC	0.006 (0.005-0.008)	Beta (61.1, 10120.7)	[28]
**Cost inputs (US$)**			
Drug costs			
PEG-RBV for 24 weeks	3609 (2707-4511)	Gamma (61.5, 58.7)	[6 38]
PEG-RBV for 48 weeks	7217 (5413- 9022)	Gamma (61.5, 117.4)	[6 38]
SOF-RBV for 12 weeks	8913 (6685-11141)	Gamma (61.5, 145.0)	[6 38]
SOF-RBV for 24 weeks	17826 (13369-22282)	Gamma (61.5, 290.0)	[6 38]
SOF-DCV for 12 weeks	12877 (9657-16096)	Gamma (61.5, 209.5)	[6 38]
SOF/VEL for 12 weeks	10518 (7888-13147)	Gamma (61.5, 171.1)	[6 38]
Monitoring costs			
Genotyping test	63	Fixed	expert opinions
Liver fibrosis test	21	Fixed	expert opinions
Routine surveillance	302	Fixed	expert opinions

Annual health states costs			
F0-F3	913 (617, 1209)	Gamma (36.6, 24.9)	[39]
F4	2599 (921, 4278)	Gamma (9.2, 282.0)	[39]
DC	5789 (3518, 8061)	Gamma (24.9, 232.0)	[39]
HCC	12220 (8788, 15653)	Gamma (48.7, 251.0)	[39]
LT	55453 (40067, 80134)	Gamma (29.4, 1883.9)	[40]
PLT	8815 (8013, 9852)	Gamma (353.2, 25.0)	[40]
Relative costs in post SVR F4	0.709 (0.592-0.855)	Normal (0.709,0.01)	[41]
Direct non-medical costs			
PEG-RBV for 24 weeks	133 (0-1451)	Gamma (0.4, 347.9)	Patient survey
PEG-RBV for 48 weeks	266 (0-2901)	Gamma (0.4, 695.9)	Patient survey
DAAs for 12 weeks	104 (0-718)	Gamma (0.6, 176.0)	Patient survey
DAAs for 24 weeks	209 (0-1436)	Gamma (0.6, 352.0)	Patient survey
Indirect costs			
PEG-RBV for 24 weeks	226 (47, 912)	Gamma (2.7, 82.1)	Patient survey
PEG-RBV for 48 weeks	451 (93, 1823)	Gamma (2.7, 164.1)	Patient survey
DAAs for 12 weeks	121 (23, 386)	Gamma (2.9, 41.4)	Patient survey
DAAs for 24 weeks	242 (47, 771)	Gamma (2.9, 82.9)	Patient survey
**Utility inputs**			
Utility of chronic disease states			
F0-F3	0.810 (0.610, 1.000)	Beta (11.8, 2.8)	[43]
F4	0.760 (0.570, 0.950)	Beta (14.0, 4.4)	[43]
DC	0.690 (0.520, 0.860)	Beta (18.9, 8.5)	[43]
HCC	0.670 (0.500, 0.840)	Beta (19.0, 9.4)	[43]
LT	0.650 (0.490, 0.810)	Beta (21.5, 11.6)	[42]
PLT	0.709 (0.530, 0.890)	Beta (16.6, 6.8)	[42]
Utility during treatment			
PEG-RBV (F0-F3)	0.700 (0.500, 0.890)	Beta (14.2, 6.1)	[44]
PEG-RBV (F4)	0.650 (0.460, 0.840)	Beta (15.1, 8.1)	[44]

DAAs (F0-F3)	0.780 (0.580, 0.970)	Beta (12.7, 3.6)	[44]
DAAs (F4)	0.730 (0.540, 0.920)	Beta (14.6, 5.4)	[44]
Utility after achieving SVR			
SVR F0-F3	0.840 (0.672, 1.000)	Beta (15.3, 2.9)	[45]
SVR F4	0.799 (0.639, 0.959)	Beta (18.5, 4.6)	[45]

DAAs, direct-acting antivirals; DC, decompensated cirrhosis; F0–F4, METAVIR fibrosis states; HCC, hepatocellular carcinoma; HCV, hepatitis C virus; LT, liver transplantation; PEG-RBV, pegylated interferon plus ribavirin; PLT, post liver transplantation; SOF-DCV, sofosbuvir plus daclatasvir; SOF-RBV, sofosbuvir plus ribavirin; SOF/VEL, sofosbuvir/velpatasvir; SVR, sustained virologic response; SVR F0–F3, patient in F0–F3 states achieving SVR; SVR F4, patient in F4 states achieving SVR.

Wei X, *et al. BMJ Global Health* 2020; 5:e003194. doi: 10.1136/bmjgh-2020-003194
